# Delivering Food Resources and Kitchen Skills (FoRKS) to Adults with Food Insecurity and Hypertension: A Pilot Study

**DOI:** 10.3390/nu15061452

**Published:** 2023-03-17

**Authors:** Rebecca L. Rivera, Mariah Adams, Emily Dawkins, Amy Carter, Xuan Zhang, Wanzhu Tu, Armando Peña, Richard J. Holden, Daniel O. Clark

**Affiliations:** 1 Division of General Internal Medicine and Geriatrics, Department of Medicine, School of Medicine, Indiana University, Indianapolis, IN 46202, USA; 2 Clem McDonald Center for Biomedical Informatics, Regenstrief Institute, Inc., Indianapolis, IN 46202, USA; 3 Eskenazi Health, Indianapolis, IN 46202, USA; 4 Department of Biostatistics and Health Data Science, School of Medicine, Richard M. Fairbanks School of Public Health, Indiana University, Indianapolis, IN 46202, USA; 5 Indiana University Center for Aging Research, Regenstrief Institute, Inc., Indianapolis, IN 46202, USA; 6 Department of Health & Wellness Design, School of Public Health, Indiana University, Bloomington, IN 47405, USA; 7 Center for Health Innovation and Implementation Science, Indiana University, Indianapolis, IN 46202, USA

**Keywords:** teaching kitchen, food insecurity, health disparities, primary care, patient-centered, chronic disease self-management education and support, hypertension, medically tailored meals, cooking skills, telehealth

## Abstract

Food insecurity affects nearly 50 million Americans and is linked to cardiovascular disease risk factors and health disparities. The purpose of this single-arm pilot study was to determine the feasibility of a 16-week dietitian-led lifestyle intervention to concurrently address food access, nutrition literacy, cooking skills, and hypertension among safety-net primary care adult patients. The Food Resources and Kitchen Skills (FoRKS) intervention provided nutrition education and support for hypertension self-management, group kitchen skills and cooking classes from a health center teaching kitchen, medically tailored home-delivered meals and meal kits, and a kitchen toolkit. Feasibility and process measures included class attendance rates and satisfaction and social support and self-efficacy toward healthy food behaviors. Outcome measures included food security, blood pressure, diet quality, and weight. Participants (*n* = 13) were on average {mean (SD)} aged 58.9 ± 4.5 years, 10 were female, and 12 were Black or African American. Attendance averaged 19 of 22 (87.1%) classes and satisfaction was rated as high. Food self-efficacy and food security improved, and blood pressure and weight declined. FoRKS is a promising intervention that warrants further evaluation for its potential to reduce cardiovascular disease risk factors among adults with food insecurity and hypertension.

## 1. Introduction

The World Health Organization defines the social determinants of health (SDOH) as “conditions in the environment…that affect a wide range of health, functioning, and quality-of-life outcomes and risks [1].” As an adverse SDOH, food insecurity—inconsistent access to a sufficient quantity of affordable, palatable, and nutritious food—is inextricably linked to poor health outcomes and disparities [2,3,4]. Among adults with food insecurity, conditions such as hypertension (HTN) are as much as two times more prevalent [5,6]. Progressive safety-net health systems have integrated SDOH screening in primary care electronic medical records and some providers refer positive cases to community resources (e.g., food pantries) [7,8]. In recent years, many of these same health systems have also implemented chronic disease self-management education and support (SMES) programs. Currently, only SMES programs for diabetes and not HTN are eligible for reimbursement under Medicare, or in 44 states under Medicaid [9,10]. However, these two healthcare innovations—addressing negative SDOH and providing HTN SMES—have not themselves been integrated, despite strong evidence that the SDOH impact SMES outcomes [5,11,12]. 

Addressing food access and affordability is critical to improving SMES outcomes, particularly among historically marginalized populations [5,13]. Notably, studies have shown that providing nutritious food aligned with a Mediterranean-style dietary pattern to adults with food insecurity could be cost-effective, and potentially more so than some common antihypertensives [14,15,16,17]. Food insecure individuals are more likely to live in communities with reduced or inconsistent access to affordable and nutritious food, as well as having limited access to lower-cost groceries and cooking tools due to budget and transportation limitations [18,19]. Both general self-efficacy (e.g., I can generally manage to solve problems) and food-related self-efficacy are lower in food-insecure individuals, as are food preparation, management, and cooking skills [20]. This translates into less nutritious food in the home and lower quality diets [12,20]. This also results in less ability to self-manage diet-sensitive chronic conditions. For example, it has been shown that food-insecure individuals have lower chronic disease self-efficacy [12,21], less responsiveness to SMES interventions [22], and one-third more hospitalizations [23]. The effectiveness of SMES programs for food-insecure populations could be improved if they directly and simultaneously addressed food insecurity [24]. 

Experiential learning, when combined with food provisions, may help sustain healthy dietary behaviors by improving individuals’ nutrition literacy and culinary skills [25,26]. Programs for teaching nutrition education to participants with incomes at or near the federal poverty line (e.g., Cooking Matters and SNAP-Education) appear to have a positive effect on participants’ skills in stretching food dollars and making healthy choices despite limited budgets [27]. Moreover, several non-randomized trials of hands-on cooking classes have shown improved food management skills, diet quality, self-efficacy, and food security [28]. One randomized controlled trial of hands-on cooking with embedded nutrition lessons demonstrated a nearly three-fold improvement in Mediterranean-style diet scores and weekly food cost savings versus traditional dietary education [25,26]. However, this trial was not conducted with food-insecure individuals nor did it include health outcomes. Studies support the hypothesis that experiential culinary learning with food provisions helps to address food insecurity. Delivered in the context of SMES, interventions have been found to improve health outcomes [29,30].

The purpose of this study was to determine the feasibility of a lifestyle intervention to concurrently address food security, nutrition literacy, and cardiovascular disease risk factors among safety-net primary care patients with food insecurity and a diagnosis of HTN. The intervention design as well as the pilot trial feasibility and behavioral and health outcomes are presented. The findings demonstrate high participation and retention rates and trends for improvement in food-related self-efficacy and skills, food security, nutrition, weight, and blood pressure.

## 2. Materials and Methods

### 2.1. Study Design

The study design was a single-arm pilot feasibility trial that took place at Eskenazi Health from 7 September 2021 to 20 January 2022 in Indianapolis, Indiana. Assessments occurred at baseline prior to the first HTN SMES class and at post-training after the conclusion of the 16-week program. 

### 2.2. Food Resources and Kitchen Skills (FoRKS) Intervention

**Figure 1:**
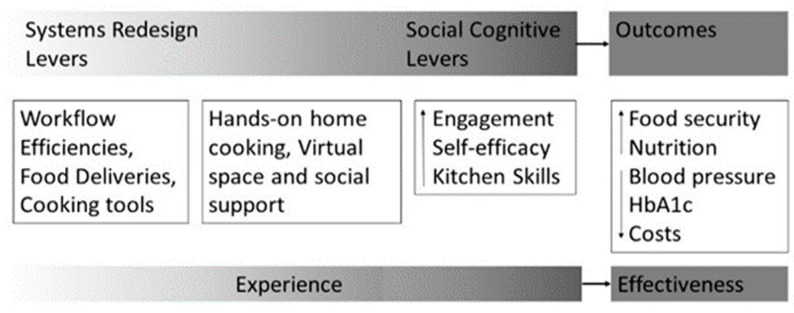
The system, experiential, and social levers, and outcomes.

A multidisciplinary team developed the dietitian-led Food Resources and Kitchen Skills (FoRKS) intervention by following a user-centered design process for Stages 0–1 of NIH’s Behavioral Intervention Development [31,32]. Together, clinical dietitians and experts in nutrition science, systems engineering, user experience design, and medical sociology produced a systems redesign of the existing HTN SMES with dietitian and patient workflow efficiencies that enabled participants to receive food and engage in experiential (i.e., hands-on) and social learning guided by the Social Cognitive Theory (SCT) [33]. As a social learning theory, SCT posits that environments designed to facilitate behavioral modeling and positive feedback from others will lead to self-efficacy gains [34]. Researchers conceptualized the system’s redesign as environmental levers through which to facilitate engagement in social and experiential learning that over time improves food-related self-efficacy and budget-conscious food-management skills (i.e., recipe selection, thrifty shopping, food planning and preparation, and cooking). In turn, these environmental levers form pathways to improved food security and health outcomes (Figure 1). 

#### 2.2.1. Hypertension (HTN) Self-Management Education and Support (SMES) Program

HTN SMES is an existing Centers for Disease Control-endorsed program offered at Eskenazi Health to provide information and skills for managing HTN. The course is led by Eskenazi Health registered dietitians, with physician, pharmacist, and health coach assistance. The standard HTN SMES structure includes five weekly classes and includes a blood pressure check and recording. The first 5 weeks of the intervention consisted of the HTN SMES program. Due to the COVID-19 pandemic, SMES class activities transitioned to live video telehealth with a typical class size of around 12 participants.

#### 2.2.2. Intervention Class Delivery

All 22 intervention classes were delivered by registered dietitians via live video telehealth conferencing. Participants received internet-enabled (via cellular data) tablet devices and tablet stands for remote participation [35]. Training on the device, including WebEx videoconference software, was completed either in person or via phone. Dietitians delivered the FoRKS intervention cooking classes from a teaching kitchen within a federally qualified health center equipped with a workstation for food preparation and cooking, and video conferencing and recording capabilities. 

#### 2.2.3. Food Delivery and Cooking Classes

Eskenazi Health’s executive chef, dietitians, and researchers developed 25 low-sodium, moderate-carbohydrate meals and recipes using culturally appropriate and familiar foods in line with the tenets of the Mediterranean diet. All meals contained less than 500 mg of sodium, and most had <15 g of sugar and <60 g of total carbohydrates per serving. Most meals included lean meats and fish as requested by the study population during recipe testing prior to study implementation, and many meals had >500 mg polyphenols. Eskenazi Health’s executive chef oversaw food procurement, meal and meal kit production, and delivery. Participants ordered from a list of medically tailored meals for home delivery on the mornings of non-cooking class days that were freshly prepared by Eskenazi Health Food Services. Up to three additional servings for meals and servings for meals and meal kits were provided based on the number of household members who routinely eat meals with the participant during the late afternoon or early evening.

Participants received a kitchen toolkit consisting of a cutting board, chef’s knife, paring knife, spatula, mixing spoon, measuring spoons, liquid and dry measuring cups, can opener, strainer, saucepan, skillet, mixing bowls, zester, vegetable peeler, and a meat thermometer to keep after the end of the study. Meal kits with fresh ingredients were delivered to participants’ homes during the mornings of cooking classes, approximately twice per week. Dietitians transitioned to cooking instruction as the SMES class component ended. Both dietitians were involved in each cooking class with one leading the cooking lesson and the other monitoring and communicating in real time with participants and the cooking dietitian. Classes 2, 4, 6, 12, and 16 did not involve cooking but focused on social learning on Food and Cooking Safety (Week 6, Class 2), Calories versus Nutrition Budget (Week 7, Class 4), Stocking a Healthy Pantry and Refrigerator (Week 10, Class 6), Seasoning and Whole Grain Review (Week 11, Class 12), and a Virtual Grocery Store Tour (Week 14, Class 16). In all other classes, the dietitians engaged participants in a discussion of the day’s recipe (ingredients, nutrition, budgeting strategies, and instructions) and cooking skills (food safety, proper tool use, and preparing ingredients—chopping/slicing, measuring, seasoning, etc.). Dietitians also provided general guidance on kitchen workspace organization. Post-cooking, there was a review of the lesson and clean-up tips. Dietitians and participants sampled the meal together virtually and shared thoughts about the meal and lesson. 

### 2.3. Participants

Potentially eligible patients were identified during clinic visits by dietitians using convenience sampling and referred to the study research manager. A research assistant telephoned potential participants for further screening, including confirmation of food insecurity. Eligible participants were adults aged 35–75 years, fluent in English, and residents of Marion County with an HTN diagnosis, last systolic blood pressure ≥120 mm Hg, Hunger Vital Sign diagnosis of food insecure, and food insecurity score ≥2 on the 18-item United States Department of Agriculture (USDA) Household Food Security Survey Module (HFSSM), indicating low or very low food security over the past 30 days [19,36,37,38]. Additionally, participants must have self-reported stable housing, independent access to a kitchen with a functional stove or hotplate, refrigerator and freezer, activity independence per functional activities questionnaire [39,40], normal cognition per six-item screener [41], and willingness to provide blood samples, use a touchscreen device, and participate via live video telehealth conferencing. 

Exclusion criteria included patients with a diagnosis or “problem list” of cognitive impairment (CI; mild CI, dementia, Alzheimer’s disease, or developmental disability), Parkinson’s disease, brain tumor, infection or surgery, serious mental illness (psychosis, schizophrenia, or bipolar disorder), or drug or alcohol abuse (alcohol consumption ≥8 drinks per week for women or ≥15 drinks per week for men), moving out of the area during the study timeline, or scheduling conflicts with the intervention. Patients currently receiving home-delivered meals through other programs (e.g., Meals on Wheels) were not eligible. FoRKS participants had access to all Eskenazi Health usual care services but received HTN SMES classes separately from non-study patients along with the additional intervention components (i.e., medically tailored, home-delivered Mediterranean-style meals and meal kits, kitchen tool kit, and live video telehealth cooking classes).

### 2.4. Data Collection

Eligible and willing patients provided informed study consent, a baseline assessment including a blood draw, and received tablet delivery with live video telehealth instruction from the research assistant who was also a licensed medical assistant. Participants connected with study personnel periodically via home visits, video conferences, or phone calls throughout the intervention setup and trial to provide feedback on their experience. Adverse events were recorded any time they became known to the study staff with action taken per the Data Safety and Monitoring Plan. Age, sex, race, ethnicity, employment status, household income, education, household size and composition, and marital status were confirmed or self-reported at baseline. The Newest Vital Sign was used to assess health literacy [42]. A mid-intervention assessment to collect blood pressure and weight and draw blood was completed in mid-December 2021. Questionnaires conducted at baseline were repeated at the post-training assessment via telephone within two weeks of completing the 16-week intervention. 

### 2.5. Process Measures

#### 2.5.1. Feasibility

Feasibility was assessed by the number of potential patients assessed for eligibility by phone, reasons for ineligibility, attendance rates, satisfaction with cooking classes, food delivery and tablet use, and participant experience. Satisfaction and experience were queried on 10–11 November 2021 and repeated on 8–9 December 2021 via individual phone calls or video conferences. Satisfaction with the cooking class, food delivery, and tablet use was measured using a five-item Likert scale ranging “not at all” to ”extremely” in response to “How happy were you with your most recent cooking class?”, “How happy were you with your most recent delivered foods?”, and “How happy were you with your most recent use of the tablet experience?”. Open-ended follow-up questions asked participants what they liked the most and least about the class and food delivery. The experience was assessed via brief, open-ended questions (e.g., “How is the class going for you?”) and a 16-item survey administered with satisfaction surveys [43]. The experience survey items asked participants how often they feel excited/interested/proud, have fun, and participate/interact with the intervention classes. Answer options included: never, hardly ever, monthly, weekly, and each day of class.

#### 2.5.2. Social Support

Social support was assessed with the Social Support and Eating Habits Survey, which has been widely used in weight loss trials and has shown good test-retest reliability (range 0.57 to 0.86) and high internal consistency (coefficient alpha 0.80 to 0.87) [44,45,46,47]. This 10-item survey captures the support or sabotage of healthy food habits by family and friends. Participants responded using a five-item Likert scale ranging from “never” to “always” over a 30-day reference period to statements about how often friends and family encouraged (e.g., remind or encourage you not to eat high-salt, high-fat, or sweet foods) or discouraged (e.g., became angry when you encouraged them to eat low-salt or low-fat foods) healthy eating habits.

#### 2.5.3. Food Self-Efficacy and Management Skills

Food self-efficacy was assessed by the nine-item Food Related Self-Efficacy Survey with part one capturing confidence and part two capturing the frequency of basic cooking, meal preparation, and meal planning skills. This survey has been shown to have good test-retest reliability (range 0.46 to 0.91) and high internal consistency (coefficient alpha 0.84 to 0.86) and to capture the change over time in response to cooking lessons [28,48]. Responses to the survey consist of a five-item Likert scale ranging from “not at all confident” to “very confident” for part one (questions 1–4) and “never” to “always” for part two (questions 5–9). 

Food management skills were assessed using the approach taken in evaluating the SNAP-Ed programs of the University of California (UC) Cooperative Extension and the University of Kentucky (UK). Specifically, we administered the Plan, Shop, Save, and Cook checklist [49]. This seven-item survey contains items, such as “How often do you plan meals ahead of time?”, “How often do you compare unit prices before buying food?”, and “How often do you shop with a grocery list?”, with a five-item Likert scale ranging from “never” to “always”. The checklist has been found to have a Cronbach’s alpha of 0.77 [49]. Both the UC and UK programs showed a 7-week pre-post improvement in program participants with this scale. The scale is available on the USDA SNAP-Ed Toolkit site [50]. 

### 2.6. Outcome Measures

#### 2.6.1. Behavioral

Food insecurity was determined using the USDA HFSSM at baseline only and the Four Domain Food Insecurity Scale (4D-FIS) at baseline and post-training assessment time points, both using a 30-day reference period [36,51]. The USDA HFSSM primarily measures how often participants experience financial strain related to insufficient food access. Response options include “never true, sometimes true, or often true” with some questions asking participants to quantify the number of days the questions affirmed were true. The survey progresses from less severe household food hardship, such as asking about anxiety over not having enough food or money for food, to more severe situations, such as decreased quality or amount of food eaten, skipping meals, and not eating for entire days among household adults and children. The 4D-FIS is a 16-item tool that expands upon the financial-focused domains of the USDA HFFSM by adding a range of experiential questions around food security in the quantitative, qualitative, psychological, and social domains. The 4D-FIS uses a four-item Likert scale ranging from “never” to “often” or, for social domain questions, “disagree a lot” to “agree”. The 4D-FIS measures individual adult food security. The 18-item USDA HSFFM measures household food security, while the food security of household adults can be calculated using the first 10 items. Both scales base food security scores on the total number of affirmative responses, therefore, a lower score is indicative of less severe food insecurity while a higher score indicates more severe food insecurity. Standard scoring and food security classification protocols are presented elsewhere [36,51]. 

Dietary data were collected with interviewer assistance using the publicly-available National Cancer Institute’s (NCI) (Rockville, MD, USA) Automated Self-Assisted 24-h Dietary Recall Tool (ASA-24) [52]. Up to two recalls were collected per participant at baseline and post-training assessment time points [53]. The research assistant conducted the first ASA-24 in person, with the participant able to view the visual prompts on the computer screen, while the second was collected via telephone between two and seven days after the first ASA-24. Diet quality was calculated from the ASA-24 data using the publicly-available NCI’s Healthy Eating Index-2015 (HEI-2015) Simple Scoring Algorithm-Per Person (Rockville, MD, USA) [54]. Units for HEI-2015 component scores are cup, ounce, or gram equivalents per 1000 kilocalories, percent of total energy, or the ratio of poly-and monounsaturated fatty acids to saturated fatty acids [55].

#### 2.6.2. Clinical

Systolic and diastolic blood pressure were measured with a digital blood pressure monitor. Body weight (pounds) was measured by the Tanita WB-800S scale (Tanita Corporation of America, Inc., Arlington Heights, IL, USA) (capacity of 660 lbs +/− 0.1 lbs).

### 2.7. Data Analysis

Feasibility outcomes for attendance rates, missed classes, satisfaction, and experience are presented as counts and averages from Likert scale scoring. Welch Two Sample T-tests were applied to detect the mean difference in outcomes between the baseline and post-training assessment time points for measures of social support, food self-efficacy, food resource management skills, food security (4D-FIS), diet quality, blood pressure, and weight. Results are reported as the means and standard deviations (mean ± SD). Significance is reported using 95% confidence intervals. Table 1 provides an overview of the study’s measurement tools. 

## 3. Results

### 3.1. Participant Characteristics 

Table 2 presents the baseline participant demographic information. A total of *n* = 13 (58.9 ± 4.5 y, 77% Female) were enrolled in the study. Twelve participants (92%) identified their race as Black or African American, and one participant identified as White. Although all participants were food insecure at baseline to meet study eligibility, about half were classified with very low food security (*n* = 7). Participants scored a mean of 3.5 ± 1.6 points on the Newest Vital Sign, indicating low to moderate health literacy. Nine participants previously completed HTN SMES: *n* = 2 in 2018, *n* = 3 in 2019, *n* = 2 in 2020, and *n* = 2 in 2021. 

### 3.2. Process Measures 

#### 3.2.1. Feasibility: Screening and Eligibility

Study dietitians identified 27 patients with systolic blood pressure ≥120 mm Hg as potentially eligible for the study. Thirteen food-insecure patients meeting eligibility requirements consented. Of the remaining patients screened for eligibility (*n* = 14), reasons for study ineligibility included food security (*n* = 6), lack of access to functional kitchen appliances (*n* = 1), schedule conflicts (*n* = 3), a health issue requiring surgery (*n* = 1), and refusal or non-response (*n* = 3). Thirteen participants completed the study baseline and post-training assessments.

#### 3.2.2. Feasibility: Attendance, Satisfaction, and Experience

The attendance rate averaged 87.1% across the 22 classes and 13 participants. Three participants attended all 22 classes, and ten participants missed an aggregated 37 classes. On average, each participant attended 19 of the 22 classes. Reasons provided for missing classes included emergency room visits (three classes), hospital admissions (two classes), illness (two classes), doctor appointments (two classes), housing insecurity (four classes), work schedule change (two classes), death of a family member (one class), and house guests (one class). A reason was not provided for 21 missed classes. Seven adverse events were recorded across six distinct participants. Table S1 (Supplementary Material) presents the number of classes missed by participants and the reason. One class was canceled (Week 10, Class 10) due to the American Thanksgiving holiday. The educational content and recipe planned for this canceled class were combined with two other classes. Appendix A provides details of the curriculum.

Participants rated satisfaction with the cooking class and food delivery extremely high. The mean score responses to how happy participants were with the cooking class and food delivery across both survey dates were 5 and 4.9, respectively. On the first satisfaction survey, participants highly rated both the cooking class and food delivery with an average score of 4.9. The cooking class reference for the first survey was Week 10, Class 9: Chicken Stir Fry with Brown Rice and Fruited Water, and the food delivery reference was Week 11, Class 11: Shrimp and Veggie Oven Packs. However, on the second satisfaction survey one month later, participants rated the cooking class higher with an average score of 5 and food delivery lower at 4.8. The cooking class reference for the second survey was Week 15, Class 17: Mix and Match Skillet Meal (participants procured their own ingredients), and the food delivery reference was Week 13, Class 15: Broccoli Alfredo and Baked Salmon. Satisfaction scores indicating extreme satisfaction with the cooking class and food delivery are presented in Table 3.

Regarding the medically tailored meals, participants reported liking that they were packaged and prepared, made to order, and fresh. Participants described the food delivery as convenient, on time, and professional. One participant who scored the cooking class with a 4 out of 5 on the first survey responded, “There wasn’t anything she [Pt 10] didn’t like about it. Would like to request her own recipes to Emily and Mariah [registered dietitians].” In reference to the food delivery that received scores of 4 on the second survey, participants reported, “[Pt 7] Doesn’t like the fish. It’s already been cooked and she likes her fish fresh.” One participant provided two ratings of 4 on the second survey but did not provide feedback about what they liked least about the cooking class or food delivery. Five participants did not like either the Mediterranean tuna salad or baked salmon dishes mainly because they do not eat or like fish. Two participants did not like the oven fajitas with chicken and beans; one reported an upset stomach and not liking the taste while the other was unsure about the red quinoa. Another reported that “She’s a picky eater—some of the food choices don’t agree with her.” Most participants provided positive feedback to open-ended satisfaction questions.

Participants rated satisfaction with tablet use experience as very high, with an average score of 4.8 across both satisfaction surveys. Ratings increased from an average of 4.7 to 4.8 from the first to second surveys. One rating of 3 was due to “difficult to get on at times” and two ratings of 4 from the same participant were due to “has trouble with it connecting” and “sometimes it messes up.” Participants provided positive responses to open-ended questions to describe their satisfaction with the tablet-use experience, such as, “ease of use”, “one button and connected”, and an “overall convenient experience”. Two participants missed the first satisfaction survey, and one participant missed the second survey.

Participants positively rated their overall experience with the intervention on the 16-item participant experience surveys administered with the satisfaction surveys. Responses revealed that participants were excited about and interested in the class, actively engaged in class activities and discussion, problem-solving, and focused on learning and participating. Out of a cumulative 23 possible responses across the two survey administrations, the response “each day of class” was provided between 17 and 23 times. Responses to questions asking about “zoning out”, a wandering mind, and pretending to or not participating in the class ranged from never to hardly never. Table 3 presents data from the participant experience surveys.

Participants found FoRKS to be a beneficial, enjoyable, and useful intervention. Thoughts participants shared include “the entire experience has just been awesome”, “recently learned how to use a cutting knife the proper way and is loving it”, “loves that everything is hands on”, and “the whole experience is so fun and they keep you captivated; it’s like a little family”. Table S2 (Supplementary Material) presents quotes from participants’ responses to the open-ended satisfaction questions. 

#### 3.2.3. Social Support, Food Self-efficacy, and Food Resource Management Skills

Family encouragement of healthy food habits decreased on average by a mean score of 2.4 (7.5) units {95% CI (−8.0, 3.0)}. Family discouragement significantly decreased on average by a mean score of 2.5 (3.5) units {95% CI (−6.5, −0.5)}. Friend encouragement increased on average by a mean score of 2.0 (6.6) units {95% CI (−2.5, 8.5)}. Friend discouragement decreased on average by a mean score of 1.2 (2.9) units {95% CI (−5.0, 1.0)}. Food self-efficacy total score improved on average by a mean score of 1.7 (5.9) units {95% CI (−2.4, 5.8)}, with significant improvement in part one by 1.8 (2.9) units {95% CI (0.5, 6.0)}, indicating increased confidence with basic cooking, meal preparation, and meal planning, and a slightly increased trend in the frequency of these behaviors shown by a 0.7 (5.3) unit improvement in part two {95% CI (−2.5, 4.0)}. Food management skills significantly improved by 2.6 (3.3) units, indicating an increase in the frequency of applying thrifty food strategies for meal planning and cooking {95% CI (0.5, 5.0)}.

### 3.3. Behavioral Outcomes: Food Security and Diet Quality 

Participants’ food security scores significantly improved by 6 (3.7) units on average from a mean total score of 7.4 (3.7) at baseline to 1.4 (1.8) units at post-training on the USDA HFSSM {95% CI (−8.5, −3.5)}. Participants improved in each of the four food insecurity domains. Participants’ mean HEI-2015 Total score increased by 3.7 (13.1) points {95% CI (−4.2, 11.6)}. Whole fruits, greens and beans, whole grains, total protein foods, fatty acids, refined grains, added sugars, and saturated fats component scores improved, while total fruits, total vegetables, dairy, seafood and plant proteins, and sodium component scores decreased. 

### 3.4. Clinical Outcomes: Blood Pressure, HbA1c, and Weight 

Mean systolic blood pressure decreased on average by 6.4 (19.0) mmHg from 141.6 (15.6) at baseline to 135.2 (18.0) mmHg at post-training. Diastolic blood pressure decreased by an average of 2.9 (13.2) mmHg. Participants lost an average of 3.2 (5.5) pounds during the 16-week intervention. Outcomes are presented in Table 4.

## 4. Discussion

The prevalence of HTN and type 2 diabetes in the United States is increasing; however, evidence for the effective management of these diseases among individuals with food insecurity is limited. Our study supported the feasibility of a lifestyle intervention with a clinic-based teaching kitchen component to address food insecurity among a low-income largely Black or African American patient population with HTN. High rates of enrollment, retention, and satisfaction with multiple intervention components provide promising support for the feasibility of the FoRKS intervention. Improvements in the social cognitive process measures give hope that this intervention will show clinically meaningful effectiveness. The pilot study findings fill gaps in the current literature by offering a potentially scalable solution to address health disparities among food-insecure populations. 

As NIH directors have recommended [56], we created an academic-clinic-patient team and applied Stages 1A and 1B of the NIH Stage Model for Behavioral Intervention Development to achieve a feasible and promising intervention that, in principle, is human-centered and culturally appropriate. For Stage 1A, we applied a co-design method to translate a theoretical framework, the SCT, into a specific lifestyle intervention in a specific context. SCT is the basis of federal nutrition education programs targeting lower-income adults [57], increasing the potential for merging and scaling our lifestyle intervention with other existing nutrition education and chronic disease self-management education and support programs. Using the well-established SCT (which in the NIH Stage Model represents the Stage 0 foundational science underpinning intervention design) ensured that the intervention had experiential learning as its cornerstone; thus, the intervention was grounded in theory about sustainable human (health) behavior [32,34].

Input from multiple stakeholders was provided during the co-design process, including participants, dietitians, researchers, and clinicians. These intervention end users worked alongside professional intervention designers and subject matter experts to collaboratively create the intervention. Advantages of the co-design method include the ability to tailor an intervention to the sociocultural environment and increase end-user commitment [58]. Additionally, the co-design method provides and encourages the engagement of historically and currently underrepresented voices, such as those represented by the participants in this study. For example, we added more lean meat and fish recipes and increased the number of cooking sessions based on participant feedback. As shown in this pilot, co-design promotes the development of interventions that are acceptable, palatable, and enjoyable by the patient and health system participants [59]. This balance in benefits across stakeholder types (i.e., participants and clinicians) is one approach to achieving what Holden et al. consider the two forms of intervention design validity: “clinical validity”, evidenced in meeting the needs of clinical best practices and workflows, and “user validity”, evidenced in meeting user needs, preferences, and values [60,61].

The co-designed intervention was then tested in a Stage 1B pilot study. This study was not designed or powered to detect significant differences in outcomes due to the intervention. Nevertheless, participants demonstrated significantly improved food security, food resource management skills, and food-related self-efficacy. Additionally, trends of improvement in systolic and diastolic blood pressure and weight were evident. 

The major strength of this study was the innovative integration of addressing food access and experiential learning through an existing and expanded HTN SMES. This structure gives repetition spaced across classes, which is a key to adult learning [62]. Each class also contained novelty via semi-structured discussions and unique recipes, which were key to engagement [63]. While preparing and cooking the recipe, the dietitians also demonstrated and shared tips and lessons. We anticipate that co-designed interventions and experiential learning will achieve higher engagement than traditional nutrition education and more significant and lasting behavior changes and health outcomes. Additional strengths included collecting validated measures for food security, food resource management, and self-efficacy. The assessment of food security status using the USDA HFSSM allows for the comparison of our results to other studies and national food security surveillance. Furthermore, the addition of the Four-Domain FIS tool provided more granular insights than the USDA HFSSM as to how participants experience food insecurity socially and psychologically, and specifics on the type of foods affected. The ASA-24 is the gold standard dietary assessment tool used in nutrition surveillance and nutrition studies; however, it has not been widely used among patient populations. This study provided a unique opportunity to simultaneously assess the feasibility of using a 24-h dietary recall in a pre-post study design among a patient population at risk for health disparities. 

The major limitation of this study was the lack of a control or comparison group and insufficient statistical power to detect differences in outcomes due to the intervention. Additionally, a lack of follow-up precluded assessing the sustainability of the intervention’s impact. We do not know whether participants maintained their improvements in food security or other health behaviors and outcomes or what adaptations would have been made or needed to sustain the intervention. The primary purpose of this pilot study was to assess the feasibility to inform a subsequent, fully powered randomized controlled trial (R01 MD017961) which will include long-term follow-up to determine the causality and effect size for health outcomes as well as cost-effectiveness. Another limitation is the dearth of evidence linking change over time in measures to health outcomes. A five-point lower systolic blood pressure or weight loss is significant if maintained [64], but changes on a self-report survey scale do not clearly map the magnitude of behavior and health outcome changes. Establishing these associations between survey measures and health outcomes is an important challenge for future studies.

In conclusion, participants were engaged and satisfied with a live video telehealth lifestyle intervention that concurrently addressed food insecurity, nutrition literacy, and cardiovascular disease risk factors through group cooking classes with experiential learning. After participating in the FoRKS intervention, the study sample of safety-net primary care patients achieved higher food security and food resource management skills. Integrating cooking classes, nutrition education, and food resources with HTN SMES has the potential to improve food security, food-related behaviors, and clinical outcomes among patients at risk for health disparities. 

## Figures and Tables

**Table 1:** Summary of the measurement tools.

Tool	Measure	Items	Administration
Screening (Baseline)			
Hunger Vital Sign [38]	Food security status	2	Interviewer (in-person)
United States Department of Agriculture Household Food Security Survey Module (USDA HFSSM) [36]	Food security status	18	Interviewer (phone)
Functional Activities Questionnaire [39,40]	Activities of daily living	10	Interviewer (phone)
Cognition Screening [41]	Cognitive impairment	6	Interviewer (phone)
The Newest Vital Sign [42]	Health literacy	2	Interviewer (in-person)
Feasibility (10–11 November 2021 and repeated on 8–9 December 2021)			
Satisfaction	Satisfaction with cooking class, food delivery, and tablet use	3	Interviewer (phone)
Experience [43]	Classroom engagement and learning	16	Interviewer (phone)
Process Outcomes (Baseline and Post-training)			
Social Support and Eating Habits Survey [45,46]	Social support or sabotage of healthy eating by family and friends	10	Interviewer (in-person)
Food-Related Self-Efficacy Survey [28,48]	Food self-efficacy	9	Interviewer (in-person)
Plan, Shop, Save, and Cook checklist [49,50]	Food resource management skills	7	Interviewer (in-person)
Behavioral Outcomes (Baseline and Post-training)			
Four Domain Food Insecurity Scale (4D-FIS) [51]	Food security status	16	Interviewer (in-person)
Automated Self-Assisted 24-h Dietary Recall Tool (ASA-24) [52]	Dietary intake	N/A	Interviewer (in-person and phone)
Healthy Eating Index (HEI)-2015 [54]	Diet quality	N/A	N/A

**Table 2:** Participant characteristics at baseline.

Characteristic	*n*	(%)
Age (years) {mean (SD)}	58.9	(4.5)
Sex		
Female	10	(77)
Male	3	(23)
Race		
Black/African American	12	(92)
White	1	(8)
USDA Household Food Security Status		
Low Food Security	6	(46)
Very Low Food Security	7	(54)
Marital Status		
Single, never married	7	(54)
Married	4	(31)
Divorced	2	(15)
Household Composition		
Lives alone	3	(23)
1 other person	2	(15)
2 or more other people	8	(62)
Children in household (<18 years)		
Yes	4	(31)
No	9	(69)
Employment status		
Employed (full or part-time)	2	(15)
Unemployed due to health status	6	(46)
Retired	3	(23)
Student	2	(15)
Total income (U.S. dollars/month)		
Less than 1500	3	(23)
Between 1500–2000	10	(77)
Education		
Less than high school diploma	4	(31)
High school diploma or GED	5	(38)
More than high school	4	(31)
SNAP participation		
Yes	9	(69)
No	4	(31)
The Newest Vital Sign {mean (SD)}	3.5	(1.6)

GED: General Education Development; USDA: United States Department of Agriculture; and SNAP: Supplemental Nutrition Assistance Program.

**Table 3:** Average score and cumulative responses of participant experience.

How Often Are These Statements True in Regard to the FoRKS Program?	Mean Score	Never	Hardly Ever	Monthly	Weekly	Each Day of Class
1. I feel excited.	4.0	-	-	-	1	22
2. I feel interested.	4.0	-	-	-	-	23
3. I feel happy.	3.9	-	1	-	-	22
4. I have fun.	3.9	-	1	-	-	22
5. I feel proud.	3.8	1	-	-	-	22
6. I get really involved in class activities.	3.9	-	-	-	2	21
7. I actively participate in class discussions.	4.0	-	-	-	1	22
8. I form new questions in my mind as I join in class activities.	3.4	2	1	1	2	17
9. I compare things I am learning with things I already knew.	3.6	-	1	1	4	17
10. I work with other participants and we learn from each other.	3.4	2	1	1	1	18
11. If I make a mistake, I try to figure out where I went wrong.	3.7	-	2	-	2	19
12. I go back over things I don’t understand.	3.5	-	3	-	2	18
13. I ask myself some questions as I go along to make sure the class make sense to me.	3.5	-	3	-	2	18
14. I am ‘zoned out’; not really thinking or participating in class.	3.8	18	5	-	-	-
15. I let my mind wander.	3.8	18	5	-	-	-
16. I just pretend like I’m participating.	3.8	19	4	-	-	-

Results are presented as the cumulative counts across two 16-item experience surveys administered on 10–11 November 2021 and repeated on 8–9 December 2021. Out of *n* = 13 participants, 2 missed the first experience survey and 1 missed the second survey, for a total of 23 responses per item. Responses to statements were scored as integers ranging from 0 to 4 (i.e., never = 0, hardly ever = 1, monthly = 2, weekly = 3, and each day of class = 4). The final three questions were reverse-scored. The survey tool was a 16-item modified Classroom Engagement Learning Inventory used to assess participant experience.

**Table 4:** The mean difference in participant outcomes from baseline to post-training assessment.

Measure (Scale Range)	Baseline		Post-Training		Difference		95% CI
	Mean	SD	Mean	SD	Mean	SD	
Process Outcomes							
Social Support for Healthy Eating (0, 25)							
Family Encouragement	11.1	8.3	8.7	7.4	−2.4	7.5	(−8.0, 3.0)
Family Discouragement	4.2	3.2	1.8	2.0	−2.5	3.5	(−6.5, −0.5)
Friend Encouragement	6.7	7.2	8.7	7.4	2.0	6.6	(−2.5, 8.5)
Friend Discouragement	3.0	2.7	1.8	2.0	−1.2	2.9	(−5.0, 1.0)
Food Self-efficacy Total (0, 45)	22.2	4.8	23.9	5.4	1.7	5.9	(−2.4, 5.8)
Part 1 (0, 20)	13.0	3.2	14.8	2.2	1.8	2.9	(0.5, 6.0)
Part 2 (0, 25)	9.2	3.3	9.9	4.1	0.7	5.3	(−2.5, 4.0)
Food Resource Management (0, 35)	14.8	4.4	17.5	4.6	2.6	3.3	(0.5, 5.0)
Behavioral Outcomes							
Four-Domain Food Security Total (0, 16)	7.4	3.7	1.4	1.8	−6.0	3.7	(−8.5, −3.5)
Quantitative (0, 3)	1.8	1.0	0.3	0.6	−1.5	1.0	(−2.5, −1.0)
Qualitative (0, 6)	2.9	1.3	0.8	1.2	−2.1	1.6	(−3.0, −1.0)
Psychological (0, 3)	1.5	1.3	0	0	−1.5	1.3	(−3.0, −2.0)
Social (0, 4)	1.3	1.0	0.3	0.6	−1.0	1.4	(−2.0, −0.5)
HEI-2015 Total (0, 100)	51.5	11.9	55.2	12.5	3.7	13.1	(−4.2, 11.6)
Total Fruits (cups) (0, 5)	2.5	2.1	2.1	2.4	−0.4	2.6	(−2.0, 1.2)
Whole Fruits (cups) (0, 5)	1.7	2.1	1.9	2.4	0.2	2.3	(−1.2, 1.6)
Total Vegetables (cups) (0, 5)	3.2	1.8	3.0	2.2	−0.3	1.8	(−1.3, 0.8)
Greens and Beans (cups) (0, 5)	1.2	2.0	1.5	2.4	0.3	2.3	(−1.1, 1.7)
Whole Grains (ounces) (0, 10)	2.3	2.9	4.3	4.1	2.0	4.1	(−0.4, 4.5)
Dairy (cups) (0, 10)	5.0	3.1	4.3	4.0	−0.7	4.7	(−3.5, 2.2)
Total Protein Foods (ounces) (0, 5)	4.6	0.7	4.9	0.2	0.3	0.8	(−0.2, 0.8)
Seafood and Plant Proteins (ounces) (0, 5)	2.5	2.5	2.0	2.3	−0.5	3.1	(−2.4, 1.4)
Fatty Acids (ratio) (0, 10)	4.9	3.9	5.2	4.1	0.2	4.8	(−2.7, 3.1)
Refined Grain (ounces) (0, 10)	7.9	2.1	9.2	1.9	1.3	2.3	(−0.1, 2.7)
Sodium (grams) (0, 10)	3.5	2.6	3.2	4.0	−0.4	4.4	(−3.0, 2.3)
Added Sugars (% energy) (0, 10)	8.7	2.2	10.0	0.4	1.1	2.4	(−0.3, 2.6)
Saturated Fats (% energy)	3.5	3.0	3.9	3.3	0.4	4.0	(−2.0, 2.8)
Clinical Outcomes							
Systolic Blood Pressure (mmHg)	141.6	15.6	135.2	18.0	−6.4	19.0	(−18.5, 5.6)
Diastolic Blood Pressure (mmHg)	88.3	10.9	85.3	11.5	−2.9	13.2	(−11.3, 5.4)
Weight (pounds)	227.2	70.4	224.0	71.6	−3.2	5.5	(−6.7, 0.3)

The Social Support for Healthy Eating (SSHE) Questionnaire queried family/friend encouragement/discouragement of healthy eating habits over the past 30 days. The Four-Domain Food Security survey reference period was the past 30 days. Units for HEI-2015 component scores are cup, ounce, or gram equivalents per 1000 kilocalories, percent of total energy, or the ratio of poly-and monounsaturated fatty acids to saturated fatty acids. mmHg: millimeters of mercury.

## Data Availability

Not applicable.

## Supplementary Materials

The following supporting information can be downloaded at: https://www.mdpi.com/article/10.3390/nu15061452/s1, Table S1: Counts of Classes Missed by Participant and Reason; Table S2: Cumulative Responses of Participant Experience.

## Appendix A

**Table A1:** Food Resources and Kitchen Skills (FoRKS) Curriculum.

Week #	Class #	Topic and Description	Curriculum	Recipes
6	1	Ready to Cook Identify needs for kitchen equipment and safe spaces for cooking; introduce knife skills	Review the cooking tool kit Knife skills during the recipe	Mediterranean tuna salad
6	2	Ready to Cook Continued Identify needs for kitchen equipment, cooking zones, and general food safety	Pictionary Cooking zones Food safety story	Not Applicable (N/A)
7	3	Food Selection and Building Ingredient Lists Using the plate method to optimize choices among food groups and plan a balanced meal	Plate method	Oven fajitas with chicken and beans
7	4	Calories vs. Nutrition Budget Concepts of energy density and nutrient density	Energy needs and energy density activities	N/A
8	5	Cooking Techniques Measuring (dry and liquid); Ggrating; stirring and mixing ingredients	Measuring practice Healthy breakfasts	Overnight oats
8	6	Stocking a Healthy Pantry and Refrigerator Focus on whole grains, produce, healthy protein, and fats	Simple substitutions Price is right	N/A
9	7	Fats: Good and Bad Fats Distinguish between different types of dietary fat and their role in the body	Artery and plaque visual activity	Healthy chicken nuggets with sweet potato and broccoli “your way”
9	8	Fats: Cooking Methods Gain an understanding of simple steps for roasting/baking; steaming; sautéing; and identifying food sources of dietary fat and opportunities for substitutions	Crisco activity Flavorful veggie “Wheel of Fortune”	N/A (broccoli was not available, so demo of create your own veggie)
10	9	Carb: Quality Carbohydrates and Fiber Cooking with whole grains and fresh produce; emphasize choosing/cleaning/forms; knife skills review Carb: Carbohydrates/Sugar Healthy beverages and knife skills review	Sugar in drinks visual activity	Chicken stir fry with brown rice and fruited water
10	10	The session was canceled due to Thanksgiving. Recipe combined with week 10 (session 9); education split among week 10 (session 9) and week 11 (session 12)	N/A	N/A
11	11	Sodium: Sodium and Sources (re-review from SMES) Cooking to create salty, bitter, sour, and umami flavor profiles	Comparing the sodium content of store-bought taco seasoning to a homemade recipe	Shrimp and veggie oven packs (uses salt-free taco seasoning)
11	12	Sodium: Seasoning Without the Salt Heart-healthy seasoning at home Heart-healthy grains: whole grain vs. refined grain	Whole grains and salt-free seasoning blends	N/A
12	13	Protein: Choosing Healthy Meats and Meat Alternatives Methods to emphasize better sources and preparation methods	Comparing the nutrition of different ground meats	Turkey and mushroom burgers
12	14	Get Ready: Cooking with Self-Purchased Ingredients Continuation of protein discussion	Lentils and plant-based proteins—recipe as a guide for discussion	Lentil and vegetable soup
13	15	Get Ready: Preparing for Grocery Shopping Outlining steps before food purchasing with pre-store planning activity	Review of shopping list infographic and options for self-acquired ingredients	Broccoli alfredo and baked salmon
14	16	Field Trip: Virtual grocery tour First-person video grocery shopping field trip- produce nutrition bang and unit price	Live demo of shopping for ingredients for the final two recipes and unit price discussion	N/A
15	17	BYOI: Bring Your Own Ingredients Shopping review	Cooking together with self-gathered ingredients	Mix and match skillet meal
16	18	BYOI: Additional Purchasing Tips Shopping review; graduation	Cooking together with self-gathered ingredients	Personal pizza
