# Supplementary material for: Delivering Food Resources and Kitchen Skills (FoRKS) to Adults with Food Insecurity and Hypertension: A Pilot Study
Source: Nutrients. 2023 Mar 17;15(6):1452. doi: 10.3390/nu15061452 (PMC10051267; doi:10.3390/nu15061452)
Supplement: Supplementary file 1 [file nutrients-15-01452-s001.zip › nutrients-2249858-supplementary.pdf]

**Supplemental Table S1.** Counts of Classes Missed by Participant and Reason

| No. Missed Classes, *n* | Pt ID | No reason | Doctor | Illness | ER visit | Housing issues | Family death | Work conflict | Hospital | House guests | Totals |
|---|---|---|---|---|---|---|---|---|---|---|---|
| 0, *n*=3 | 3 | - | - | - | - | - | - | - | - | - | **0** |
| | 5 | - | - | - | - | - | - | - | - | - | **0** |
| | 12 | - | - | - | - | - | - | - | - | - | **0** |
| 1, *n*=2 | 4 | 1 | - | - | - | - | - | - | - | - | **1** |
| | 7 | - | - | - | 1 | - | - | - | - | - | **1** |
| 2, *n*=4 | 1 | 1 | 1 | - | - | - | - | - | - | - | **2** |
| | 2 | 1 | - | - | 1 | - | - | - | - | - | **2** |
| | 11 | 1 | - | - | - | - | - | - | - | 1 | **2** |
| | 13 | 2 | - | - | - | - | - | - | - | - | **2** |
| 4, *n*=1 | 9 | 3 | - | - | - | - | - | - | 1 | - | **4** |
| 6, *n*=2 | 8 | 2 | - | 2 | - | - | 1 | 1 | - | - | **6** |
| | 10 | 3 | 1 | - | 1 | - | - | 1 | - | - | **6** |
| 11, *n*=1 | 6 | 7 | - | - | - | 4 | - | - | - | - | **11** |
| **Totals** | | **21** | **2** | **2** | **3** | **4** | **1** | **2** | **1** | **1** | **37** |

Results are provided as counts per participant (*n*=13) sorted by total number of missed classes in ascending order. Column totals are counts per reason. Row totals are counts per participant. Participants missed a total of 37 classes. ER, emergency room

**Supplemental Table S2:** Summary of Participant Perspectives on Intervention Components: Cooking Classes, Food Delivery, and Tablet Experience

| Theme | Subtheme | Representative Quote (s) |
|---|---|---|
| Cooking Classes | Facilitators | "To get acquainted on how to do some things that she hasn't had much experience with. It's exciting for her to learn some things she didn't know. The taste wasn't bad either. Has taught her that she doesn't need much very seasoning and she's even lost a few pounds." (Pt 1)<br>"It was something real simple, and really easy to do and understand. She likes the ingredients are supplied." (Pt 2)<br>"The networking together. Loves it- likes to see how people do different things in the kitchen. The meals are different than what he is used to and realizes it is good food. Loves to cook so he loves every minute of it. He likes the food- it was pretty marvelous." (Pt 3)<br>"It's fun and he enjoys it! He enjoys the food too! He likes learning how to cook and learning how to eat right. He got to eat." (Pt 4)<br>"Cooking together as a group. Just cooking and enjoying the participants." (Pt 5)<br>"Every class she's had has been alright with her. Different recipes and the ingredients- likes that they are natural ingredients." (Pt 6)<br>"I'm loving the class. Typically doesn't like mushrooms, spinach, or ground turkey but she loved it and plans to make it again for dinner tonight." (Pt 7)<br>"Is learning how to eat healthy- teaching her how to assess calories, and portion control. She loves the group and the teachers." (Pt 8)<br>"He's getting a lot of useful information. Some stuff he already knew, but other stuff is new to him." (Pt 9)<br>"It was fun- learning new stuff and preparing food differently from what she usually uses. They're teaching her how to cook better w healthier options, but the food is actually tasting good." (Pt 10)<br>"She loves the group." (Pt 11)<br>"All of the goodies she gets. We are in a group and are able to talk to each other and everyone is caring." (Pt 12) |
| | Barriers | "There wasn't much she didn't like - the standing is an issue but she got through it. She plans to get a stool to accommodate her needs. Wasn't able to participate. Wasn't able to participate very much because of timing and what she has going on." (Pt 1)<br>"Just difficult to do with the tablet and wifi, signal problems. If they walk away from microphone on the tablet it is hard to hear." (Pt 9)<br>"She's a picky eater- some of the food choices don't agree w her." (Pt 12) |
| Food Delivery | Facilitators | "Some was pulled pork, some was a chicken salad, this delivery was nice and light." (Pt 1)<br>"The fact that it was already prepared. She likes that she can choose what she gets." (Pt 2)<br>"Perfect and right on time. They're just different foods that he normally buys. Usually doesn't prepare the foods the way it comes and realizes he likes it. He liked the soup because of his current teeth issues." (Pt 3) |

| | | |
|---|---|---|
| | | "I like that they are delivered and the containers, it keeps the food fresh. He was on time, he's respectful. He likes that he can pick his own foods and switch them out if he didn't like them." (Pt 4)<br>"Haven't had any issues thus far. They arrive on time, and they are nice and fresh when they get here." (Pt 5)<br>"It's ok- the driver has a professional manner. They were delivered fresh." (Pt 6)<br>"Convenient and doesn't have to think about it. Everyone enjoyed the chicken fajitas." (Pt 7)<br>"Gives her opportunity to switch it out. They gave her what she requested." (Pt 8)<br>"It's already prepared. Microwaveable and easy to prepare. The portions look small and was worried they wouldn't be enough food, but he does feel full after he eats them." (Pt 9)<br>"Liked how they were packaged and prepared. The batch she received is everything she likes it. Her husband actually ate it too." (Pt 10)<br>"She likes that it is on time and the packaging is good." (Pt 11)<br>"It was on time. Something that she can pick out that she likes. She likes they accommodate things to her liking." (Pt 12)<br>"Likes that it is heart healthy." (Pt 13) |
| | Barriers | "The Mexican food hurts her belly and she didn't really like the taste…some of the food she didn't particularly like, but she didn't allow her to give up on it." (Pt 1)<br>"She said one time the delivery guy, just left it on her porch without it being in the cooler. Mariah handled that…the Mexican skillet- isn't sure about the red quinoa." (Pt 6)<br>"This was the first one where she really enjoyed all of the food. Doesn't like the fish. It's already been cooked and she likes her fish fresh." (Pt 7)<br>"Some of the stuff she doesn't like." (Pt 8)<br>"Not necessarily everything he likes but wouldn't give it a bad rating." (Pt 9)<br>"Don't do the fish. Doesn't like baked fish." (Pt 10)<br>"She didn't like the Mediterranean dish." (Pt 11)<br>"She doesn't eat fish and she's a picky eater." (Pt 12) |
| Tablet Experience | Facilitators | "All coming together- being able to communicate with the other participants. Nothing she can think of. She has learned how to use it and she likes learning about it." (Pt 1)<br>"She only uses it for this and is comfortable using it." (Pt 2)<br>"He loves it. Excellent- hasn't run into any issues. It is easy to navigate. He's so happy with it- that he wants to go out and buy one. He can't believe the battery life it has on it." (Pt 3)<br>"He loves it and it's very convenient. He likes it- because it is very interesting." (Pt 4)<br>"It's easy to use. Pretty much no problems signing on unless there are issues with the WIFI." (Pt 5)<br>"The program is easy to get into. She likes the way it is setup. The platform set up is great. She wishes they could keep the tablet." (Pt 6) |

| | | |
|---|---|---|
| | | "It's perfect. She just has to push 1 button and she is connected. It's easy and she can get right to the class." (Pt 7)<br>"It's fine. She's able to see everyone." (Pt 8)<br>"Loves the stand." (Pt 9)<br>"It's bigger to see into and she doesn't have to hold it, it's mobile. It's like her phone and she can see things better. She actually wants to keep it." (Pt 10)<br>"It has it's own internet. She loves it she just loves it. She can get on it anywhere. She likes that she can mute it." (Pt 12)<br>"She wants to keep it." (Pt 13) |
| | Barriers | "Difficult to get on at times." (Pt 1)<br>"Keeps forgetting to charge it." (Pt 7)<br>"Sometimes she has trouble with it connecting. Sometimes it messes up." (Pt 8)<br>"Unfamiliar with it bc he's not used to Apple products." (Pt 9)<br>"Needs to keep her daughter off of it." (Pt 10)<br>"It's not mine. She had a sound problem one day where she had to use her phone." (Pt 12) |
